# Hints for the Bath

**Published:** 1892-08

**Authors:** 


### HINTS FOR THE BATH.

Put to a cup of sea salt, a half-ounce of camphor, a half-ounce of ammonia in a quart bottle ; fill the bottle with hot water and let it stand twenty-four hours ; then, when prepared to bathe with a sponge, put a teaspoonful of this mixture, well shaken, into your basin. A surprising quanity of dirt will come from the cleanest skin. The ammonia cleanses, and the camphor and the sea salt impart a beneficial effect which cannot be exaggerated.

